# Real-world evidence regarding the growth of very premature infants with small for gestational age after birth: a multicenter survey in China

**DOI:** 10.1186/s12887-023-04245-1

**Published:** 2023-08-31

**Authors:** Xue-Rong Huang, Wei Shen, Fan Wu, Jian Mao, Ling Liu, Yan-Mei Chang, Rong Zhang, Xiu-Zhen Ye, Yin-Ping Qiu, Li Ma, Rui Cheng, Hui Wu, Dong-Mei Chen, Ling Chen, Ping Xu, Hua Mei, San-Nan Wang, Fa-Lin Xu, Rong Ju, Zhi Zheng, Xin-Zhu Lin, Xiao-Mei Tong, the Chinese Multicenter EUGR Collaborative Group, Xinzhu Lin, Qianxin Tian, Qiliang Cui, Yuan Yuan, Ling Ren, Bizhen Shi, Yumei Wang, Jinghui Zhang, Yan Zhu, Chao Chen, Jingjing Zou, Yuhuai Li, Baoyin Zhao, Shuhua Liu, Ying Xu, Wenli Zhou, Zhiyong Liu, Jinzhi Gao, Jing Liu, Cong Li, Chunyan Yang, Yayu Zhang, Sile Hu, Zuming Yang, Zongtai Feng, Er-Yan Meng, Li-Hong Shang, Shaoping Ou, Gui-Nan Li, Long Li, Zhe Zhang, Fei Bei, Chun Deng, Ping Su, Ling-Ying Luo, Xiao-Hong Liu, Li-Jun Wang, Shu-Qun Yu

**Affiliations:** 1 Department of Neonatology, Women and Children’s Hospital, School of Medicine, Xiamen University, Xiamen, 361003 Fujian China; 2 Xiamen Key Laboratory of Perinatal-Neonatal Infection, Xiamen, 361003 Fujian China; 3 Department of Neonatology, the Third Affiliated Hospital of Guangzhou Medical University, Guangzhou, 510150 Guangdong China; 4 Department of Pediatrics, Shengjing Hospital of China Medical University, Shenyang, Liaoning, 110000 China; 5 Department of Neonatology, Guiyang Maternal, and Child Health Hospital Guiyang Children’s Hospital, Guiyang, Guizhou 550002 China; 6 Department of Pediatrics, Peking University Third Hospital, Beijing, 100191 China; 7 Department of Neonatology, Children’s Hospital of Fudan University, Shanghai, 201102 China; 8 Department of Neonatology, Guangdong Province Maternal and Children’s Hospital, Guangzhou, 510030 Guangdong China; 9 Department of Neonatology, General Hospital of Ningxia Medical University, Yinchuan, 750001 Ningxia China; 10 Department of Neonatology, Children’s Hospital of Hebei Province, Shijiazhuang, 050031 Hebei China; 11 Department of Neonatology, Children’ Hospital of Nanjing Medical University, Nanjing, 210000 Jiangsu China; 12 Department of Neonatology, the First Hospital of Jilin University, Changchun, Jilin, 130000 China; 13 Department of Neonatology, Quanzhou Maternity and Children’s Hospital, Quanzhou, 362000 Fujian China; 14 Department of Pediatrics, Tongji Hospital, Tongji Medical College, Huazhong University of Science and Technology, Wuhan, 430000 Hubei China; 15 Department of Neonatology, Liaocheng People’s Hospital, Liaocheng, 252000 Shandong China; 16 Department of Neonatology, the Affiliate Hospital of Inner Mongolia Medical University, Hohhot, 010010 Inner Mongolia China; 17 Department of Neonatology, Suzhou Municipal Hospital, Suzhou, 215002 Jiangsu China; 18 Department of Neonatology, The Third Affiliated Hospital of Zhengzhou University, Zhengzhou, 450052 Henan China; 19 Department of Neonatology, Chengdu Women’ and Children’s Central Hospital, School of Medicine, University of Electronic Science and Technology of China, Chengdu, 611731 Sichuan China; 20 Nutritional Committee of Neonatology Branch of Chinese Medical Doctor Association, National Multicenter EUGR Collaborative Group, Beijing, 100191 China

**Keywords:** Extrauterine growth retardation, Extremely premature infants, GV, Nutrition, Small for gestational age, Z score

## Abstract

**Background:**

To analyze the real-world growth pattern of very premature infants (VPI) with small for gestational age (SGA) after birth by using the ΔZ value of weight at discharge.

**Methods:**

The clinical data were collected from 28 hospitals in China from September 2019 to December 2020. They were divided into the EUGR(Extrauterine Growth Restriction) and the non-EUGR group according to the criterion of ΔZ value of weight at discharge < –1.28.

**Results:**

This study included 133 eligible VPI with SGA. Following the criterion of ΔZ value, the incidence of EUGR was 36.84% (49/133). The birth weight, the 5-min Apgar score, and the proportion of male infants in the EUGR group were lower (*P* < 0.05). The average invasive ventilation time, cumulative duration of the administration of antibiotics, blood transfusion time, blood transfusion ratio, and total days of hospitalization were significantly higher in the EUGR group (*P* < 0.05). In the EUGR group, several factors exhibited higher values (P < 0.05), including the initiation of enteral feeding, the volume of milk supplemented with human milk fortifier (HMF), the duration to achieve complete fortification, the cumulative duration of fasting, the duration to achieve full enteral feeding, the length of parenteral nutrition (PN), the number of days required to attain the desired total calorie intake and oral calorie intake, as well as the age at which birth weight was regained. The average weight growth velocity (GV) was significantly lower in the EUGR group (*P* < 0.001). The incidences of patent ductus arteriosus with hemodynamic changes (hsPDA), neonatal necrotizing enterocolitis (NEC) stage≥ 2, late-onset sepsis (LOS), and feeding intolerance (FI) in the EUGR group were higher (*P* < 0.05). Multivariate logistic regression analysis showed that birth weight, male, and GV were the protective factors, while a long time to achieve full-dose fortification, slow recovery of birth weight, and NEC stage ≥2 were the independent risk factors.

**Conclusion:**

SGA in VPI can reflect the occurrence of EUGR more accurately by using the ΔZ value of weight at discharge. Enhancing enteral nutrition support, achieving prompt and complete fortification of breast milk, promoting greater GV, reducing the duration of birth weight recovery, and minimizing the risk of NEC can contribute to a decreased occurrence of EUGR.

**Trial registration:**

CHICTR, ChiCTR1900023418. Registered 26/05/2019, http://www.chictr.org.cn.

## Background

With an increase in the understanding of short-term and long-term health-influencing factors that affect SGA, the perinatal medical community has focused on the prevention and management of nutrition of SGA infants. Regarding the incidence of SGA, China (6.5% incidence) ranks fifth globally (16% incidence) [1]. In 2016, the WHO defined SGA as a newborn whose birth weight is below the 10^th^ percentile of the birth weight for infants of the same sex of the same gestational age or whose Z-value of birth weight is < –1.28. The Fenton growth curve (2013) [2] is used for the diagnosis of SGA. SGA can be divided into premature SGA, full-term SGA, and overdue SGA, among which premature SGA is affected by intrauterine growth retardation and immature gestational age. The risk of early complications after birth and perinatal death increases, and it can also lead to many long-term complications such as adult cardiovascular diseases, insulin resistance, and neurocognitive dysfunction, which increases the burden on society and families.

Guellec et al. [3] established a correlation between postnatal growth impairment in infants with SGA and cognitive deficits and learning difficulties. This finding has been supported by additional studies. For example, in their publication in the Journal of Pediatrics, Kerstjens et al. [4] discovered a connection between postnatal growth impairment in SGA infants and delayed intellectual development and learning difficulties. Euser et al. [5] also identified an association between postnatal growth impairment in SGA infants and behavioral and emotional problems. These research outcomes emphasize the significance of monitoring and intervening in the postnatal growth of SGA infants to mitigate the occurrence of extrauterine growth restriction (EUGR) and enhance their neurodevelopment and growth. Currently, there is no international consensus regarding the optimal postnatal growth pattern for preterm SGA infants. It is imperative to closely monitor the growth pattern of preterm newborns to detect any deviations from the norm. Early and appropriate catch-up growth plays a beneficial role in the physical growth and neurodevelopment of SGA children. Therefore, it is essential to develop reliable methods for accurately identifying infants with genuine extrauterine growth restriction, comprehending the factors influencing the occurrence of EUGR, and providing adequate and appropriate nutrition. These measures are crucial for ensuring successful catch-up growth [6, 7].

However, as a consequence of intrauterine growth retardation, SGA infants exhibit slow growth and development. Consequently, it becomes challenging for the growth and development parameters of SGA infants to reach the 10^th^ percentile value for the corresponding gestational age upon discharge. Thus, it takes a long time to complete the catch-up growth [8]. Therefore, the incidence of extrauterine growth retardation (EUGR) in SGA infants is significantly higher than the incidence of EUGR in non-SGA infants. Many studies have reported that SGA is an independent risk factor for EUGR [9, 10].

EUGR is related to intrauterine growth retardation (IUGR). Studies generally refer to the Fenton growth curve (2013) and define the 10^th^ percentile of the weight, height, and head circumference at the corrected gestational age of 36 weeks or at discharge as EUGR and the 3^rd^ percentile below the growth curve as severe EUGR. By this cross-sectional definition, the incidence of EUGR in SGA is 87.6% ~ 98.5% [9, 11], which is significantly higher than 44.44% in non-SGA [9]. Some researchers have suggested that the occurrence of EUGR in SGA is a continuation of intrauterine growth retardation but not “real EUGR” [12]. Therefore, the percentile (*P*-value) of the Fenton growth curve cannot reflect the growth pattern of SGA after birth. To better reflect the growth status of premature infants after birth, Simon et al. [13] suggested that the change in the Z scores between the weight at discharge and birth weight (ΔZ value) should be be used as part of the longitudinal definition to evaluate EUGR. The Z-score indicates how far the infant’s weight and height are from the 50^th^ percentile or the median of the reference growth charts for infants of the same age and sex, i.e., Z value = (measured value-average value of the same gestational age and gender)/standard deviation of this gestational age and gender). Studies have shown that dynamic longitudinal definition is more effective than cross-sectional definition in predicting adverse neurodevelopmental outcomes at a 2-year follow-up [14]. Furthermore, longitudinally defined EUGR is associated with weight and head circumference deficits at 24–30 months of age [15]. Therefore, the longitudinal definition is superior to the cross-sectional definition in predicting long-term outcomes in preterm infants, and whenever feasible, it should be the preferred method for diagnosing EUGR. Therefore, the ΔZ value might be more suitable for analyzing the extrauterine growth of individuals after birth [13]. We conducted a national prospective multicenter study in China to analyze the real-world incidence of EUGR and risk factors that affect very premature infants (VPI) in SGA, based on the ΔZ value of weight.

## Objective and methods

### Study population

This study encompassed a prospective survey conducted across multiple centers from September 2019 to December 2020. Data for the study were gathered from 28 tertiary hospitals located in seven regions of China, including the northeastern, northern, eastern, central, southern, northwestern, and southwestern regions. The protocol was approved by the Ethics Committee of Women and Children’s Hospital affiliated with Xiamen University/Xiamen Maternity and Child Health Care Hospital (KY-2019–016), and the study was registered in the Chinese Clinical Trials Registry (http://www.chictr.org.cn) with the registration number ChiCTR1900023418. Prior to participating in the study, written informed consent was obtained from the parents, ensuring their full understanding and agreement. The methodology employed in this study adhered to the applicable guidelines and regulations, ensuring its compliance with ethical standards.

We collected the clinical data of VPI with SGA hospitalized in the above mentioned multicenters. Inclusion criteria: ① SGA; ② Birth gestational age < 32 weeks; ③ Hospitalization time > 2 weeks; ④ Admission within 24 h after birth. Exclusion criteria: ① Congenital malformation or genetic metabolic disease; ② Death, interruption of treatment, or automatic discharge during hospitalization; ③ Incomplete data.

### The VPI with SGA were divided into the EUGR and non-EUGR groups

A change in the Z-score (△Z value) of weight by more than 1.28 between two points (discharge and birth) was considered to be EUGR, and a change in the Z-score (△Z value) of weight by less than 1.28 was considered to be non-EUGR [16].

### Methods

Using a unified questionnaire, perinatal data of VPI with SGA were collected (gestational age at birth, Z value of physical indices at birth, sex, delivery mode, multiple births, prenatal glucocorticoid administration, and the 5-min Apgar score), maternal and pregnancy complications (gestational hypertension and gestational diabetes), growth and nutritional status during hospitalization [maximum weight loss, the age of recovering birth weight, the average weight gain velocity (GV), the ΔZ-value of physical indices at discharge, start time of enteral feeding, the age of reaching total enteral nutrition, cumulative fasting days, breast milk volume after the addition of human milk fortifier (HMF) and days needed for full fortification, the age of reaching the standard of oral calorie, cumulative calorie intake in the first week of hospitalization, cumulative dose of amino acids and fat milk in the first week of hospitalization, the duration of parenteral nutrition (PN)],main treatment conditions (invasive mechanical ventilation time, total oxygen consumption time, the use rate of postnatal hormones, cumulative duration of antibiotics used, hospitalization time) and main complications during hospitalization [neonatal respiratory distress syndrome (NRDS), early-onset sepsis (EOS), feeding intolerance (FI), patent ductus arteriosus with hemodynamic changes (hsPDA), neonatal necrotizing enterocolitis (NEC) ≥ stage 2, bronchopulmonary dysplasia (BPD), late-onset sepsis (LOS), grade III-IV intraventricular hemorrhage (IVH), periventricular leukomalacia (PVL), parenteral nutrition-associated cholestasis (PNAC), retinopathy of prematurity (ROP) requiring intervention, metabolic bone disease of prematurity (MBDP), EUGR], and other clinical data were also collected.

### Definition or diagnostic criteria of related diseases

(1) SGA is a newborn whose birth weight is lower than the 10^th^ percentile of the birth weight of a newborn of the same sex, and gestational age or whose birth weight Z value is < –1.28; (2) The EUGR evaluation criteria refer to the Fenton growth curve [2] published in 2013. ① The evaluation criteria for percentile (*P* value) were as follows: VPI with a weight below the 10^th^ percentile, based on the 2013 Fenton growth curve, at 36 weeks of corrected gestational age or upon discharge;② ΔZ value evaluation criteria: △Z value of weight = (Z value of weight at 36 weeks of corrected gestational age or during discharge-Z value of birth weight); EUGR is defined as weight ΔZ value < –1.28 [16]; (3) BPD is defined as a newborn with persistent oxygen dependence for ≥ 28 days after birth [17]; (4) EOS and LOS diagnostic criteria [18] refer to the consensus of experts on the diagnosis and treatment of neonatal sepsis (2019 edition); (5) FI diagnostic criteria [19]: the stomach residue exceeds 50% of the previous feeding amount, accompanied by vomiting and/or abdominal distension; the feeding plan fails, including reduced, delayed, or interrupted enteral feeding; (6) Diagnostic criteria of MBDP: refers to the consensus of clinical management experts of metabolic bone disease in premature infants (2021) [20]; (7) NEC ≥ stage 2: was defined as Bell stage≥2 [21]; (8) Diagnostic criteria of hsPDA: PDA catheter diameter > 1.5 mm, accompanied by heart murmur, tachycardia, rapid respiration, increased pulse pressure, hypotension; (9) The complications such as NRDS, IVH ≥ stage 3, PVL, PNAC, and ROP need intervention; refer to the diagnostic criteria [22] in *Practical Neonatology (5*^*th*^* Edition).*

### Definition of enteral nutrition

(1) Start time of enteral feeding (h): the time to start oral feeding/nasal feeding of breast milk or formula milk after birth (excluding colostrum oral care); (2) Total enteral feeding time (d): the time required for oral milk intake to reach 150 mL/kg/d; (3) Time for total and oral calorie intake to reach the target: the recommended calorie intake standard was 110 kcal/(kg·d). (4) Mean GV [g/(kg·d)]: [1,000 × ln (Wn/W1)]/(Dn-D1) after regaining birth weight. In this formula, Wn indicates weight (g) at discharge, W1 indicates birth weight (g), Dn indicates the length of hospital stay (day), and D1 indicates the time to regain birth weight (day) [23].

### Statistical analysis

Statistical analysis was conducted using the SPSS 22.0 software. Measurement data that exhibited a normal distribution were reported as mean ± SD, and a comparison between groups was performed using independent-samples t-tests. Non-normally distributed quantitative data were presented as the median and interquartile ranges, and the Mann–Whitney U test was conducted for comparison between groups. The count data were presented as the number and rate of cases, and the Chi-squared test or Fisher’s exact test was conducted for comparison between groups. Variables that demonstrated a significance level of *P* < 0.05 in the single-factor analysis were selected for inclusion in the multivariate analysis. A stepwise approach was employed to screen these variables by constructing a multivariate logistic regression model, with a significance level (α) set at 0.05. All differences among and between groups were considered to be statistically significant at *P* < 0.05.

## Results

### The incidence of EUGR

During the study period, data on 2,600 VPI were collected. Of these, 86 cases were excluded due to incomplete information about the mother and the infants, 2,381 cases of non-SGA in VPI were excluded, and finally, 133 VPI with SGA were included in the study, who were evaluated based on the Fenton curve. The birth weight between the EUGR and the non-EUGR groups was not significantly different (*P* = 0.881), but the weight of the EUGR group at discharge was significantly lower (0.31 vs. 16.32, *P* = 0.012). The incidence of EUGR in VPI with SGA was determined to be 98.50% (131 out of 133 cases) based on the weight, 89.47% (119 out of 133 cases) based on the Length and 81.20% (108 out of 133 cases) based on the Head circumference of infants at 36 weeks of corrected gestational age or at discharge, using the 10^th^ percentile of the 2013 Fenton growth curve and According to the standard ΔZ value of the weight, the Z scores of the birth and discharge weights of the EUGR group were lower than those in the non-EUGR group (-1.58 vs. –1.49, *P* = 0.017; –3.54 vs. –2.21, *P* < 0.001). Additionally, the data for head circumference and body length were as follows: head circumference -1.52 vs. -0.52, *P* < 0.001; body length -2.48 vs. -1.51, *P* < 0.001). For ΔZ value of weight at discharge < –1.28, there were 49 cases in the EUGR group and 84 cases in the non-EUGR group, and the incidence of EUGR was 36.84% (49/133 cases);As for length, the ΔZ value was observed in 35 cases (26.32%), and for head circumference, the ΔZ value was observed in 20 cases (15.04%). see Table 1.

**Table 1:** Comparison of the incidence of EUGR evaluated by the *p*-value and the ΔZ value at discharge between the EUGR and the non-EUGR groups

EUGR standard	Non-EUGR	EUGR	*t/Z*	*P*
**Evaluate with** ***P*-value**				
**Weigh** ***P*-value [n (%)]**	2(1.50)	131(98.50)		
Percentile at birth [M (Q1, Q3)]	6.62(4.65,8.25)	6.36(5.85,06.87)	-1.49	0.881
Percentile at Discharge [M (Q1, Q3)]	16.32(12.57,20.08)	0.31(0.06,0.98)	-2.42	0.012
**Length** ***P*-value**	14(10.53)	119(89.47)		
Percentile at birth [M (Q1, Q3)]	12.22(4.47,17.06)	2.87(0.69,8.91)	-2.90	0.004
Percentile at Discharge [M (Q1, Q3)]	17.26(12.31,20.8)	0.31(0.01,2.09)	-6.01	< 0.001
**Head circumference *P*-value**	25(18.80)	108(81.20)		
Percentile at birth [M (Q1, Q3)]	13.8(5.17,31.35)	6.4(1.7, 17.96)	-2.13	0.021
Percentile at Discharge [M (Q1, Q3)]	20.14(15.27,27.95)	1.87(0.41,5.23)	-8.59	< 0.001
**Evaluate with Δ z < -1.28**				
**Weight Δ z value [n (%)]**	84(63.16)	49(36.84)		
Z score at birth [M (Q1, Q3)]	-1.49(-1.61, –1.37)	-1.58(-1.85, –1.43)	-2.39	0.017
Z score at Discharge [x ± s]	-2.21 ± 0.55	-3.54 ± 0.69	12.17	< 0.001
**Length Δ z value [n (%)]**	98(73.68)	35(26.32)		
Z score at birth [M (Q1, Q3)]	-1.55(-1.13,-2.32)	-1.88(-1.34, -1.28)	-1.78	< 0.001
Length score at Discharge[M (Q1, Q3)]	-2.00(-2.83,-1.56)	-3.84(-2.73, -4.49)	-6.02	< 0.001
**Head circumference Δ z value [n (%)]**	113(84.96)	20(15.04)		
Z score at birth [M (Q1, Q3)]	-0.52(-0.95,-0.20)	-1.52(-1.01, -2.13)	-4.53	< 0.001
Z score at Discharge [M (Q1, Q3)]	'-1.51(-2.1,-0.94)	-2.48(-3.11,-2.08)	-4.04	< 0.001

*EUGR *Is extrauterine growth retardation, *SGA* Is smaller than gestational age, *VPI* Is very premature infants

### General information and main treatment of VPI with SGA during the perinatal period

Following the criterion of ΔZ of weight < –1.28, the birth weight, the 5-min Apgar score, and the incidence of male infants in the EUGR group were lower than those in the non-EUGR group (*P* < 0.05 for all parameters). Significant differences (*P* < 0.05) were observed between the EUGR group and the non-EUGR group in several parameters. These included a higher average duration of invasive ventilation, cumulative antibiotic use, number of blood transfusions, blood transfusion ratio, and total hospitalization days in the EUGR group. The gestational age, pregnancy hypertension, gestational diabetes, delivery mode, multiple births, the rate of administration of postnatal hormones, noninvasive mechanical ventilation time, and nasal catheter oxygen supply time were not significantly different between the EUGR and the non-EUGR groups (*P* > 0.05); see Table 2.

**Table 2:** Comparison of the general perinatal information and main treatment of VPI with SGA between the EUGR and non-EUGR groups

Variable	Non-EUGR *n* = 84	EUGR *n* = 49	*t/Z/χ*^*2*^	*P*
Male [n (%)]	46(75.41)	30(41.67)	15.353	< 0.001
Birth age Week [*x* ± *s*]	30.58 ± 1.40	30.23 ± 1.43	1.372	0.172
Gestational.age.At.discharge	39.00(38.00, 40.00)	38.00(37.00, 38.25)	-1.123	< 0.001
Birth weight g [*x* ± *s*]	976.50 ± 176.35	854.92 ± 170	3.886	< 0.001
Cesarean section [n (%)]	78(92.86)	46(93.88)	/	> 0.999
Use rate of postnatal hormones [n (%)]	68(80.95)	44(89.8)	2.087	0.352
Pregnancy hypertension [n (%)]	47(55.95)	34(69.39)	2.346	0.126
Gestational diabetes [n (%)]	8(9.52)	4(8.16)	/	> 0.999
Multiple births [n (%)]	23(27.38)	20(40.82)	2.553	0.111
5 min Apgar [M (Q1, Q3)]	9(8,10)	8(7,9)	-2.52	0.012
Invasive ventilation time d [M (Q1, Q3)]	0(0,2.50)	2(0,7)	2.934	0.003
Noninvasive ventilation time d [M (Q1, Q3)]	18.5(7.5,29)	19(9,32)	0.74	0.459
Oxygen use time of nasal catheter d [M (Q1, Q3)]	9.65(4,19)	13(4,25)	1.508	0.132
cumulative duration of antibiotics use d [M (Q1, Q3)]	12.5(6.50,17.50)	16(10,25)	2.54	0.011
Frequency of blood transfusion d [M (Q1, Q3)]	1(0.5,20)	3(1,6)	3.656	< 0.001
Blood transfusion ratio [n (%)]	61(72.62)	43(87.76)	4.158	0.041
Total hospitalization days d [x ± s]	53.82 ± 17.39	69.08 ± 16.92	-4.929	< 0.001

Remarks:/: Fisher’s accurate test, no such value

*EUGR* Is extrauterine growth retardation, *SGA* Is smaller than gestational age, *VPI* Is very premature infants

#### Nutritional status of VPI with SGA in the hospital

Following the criterion of ΔZ of weight < –1.28, the start time of enteral feeding, the amount of milk added with HMF, the time to reach full fortification, the cumulative fasting time, the time to reach total intestinal feeding, the duration of PN, the number of days to reach the target total calorie intake and oral calorie intake (both 110 kcal/kg/d), and the date of recovery of birth weight in the EUGR group were significantly more than those in the non-EUGR group (*P* < 0.05). GV exhibited a significantly lower value in the EUGR group compared to the non-EUGR group (*P* < 0.001). During the first week of hospitalization, there were no significant differences (*P* > 0.05) between the EUGR and non-EUGR groups in terms of accumulated amino acids, fat emulsion, accumulated calories, and maximum physiological weight loss. Please refer to Table 3 for detailed information.

**Table 3:** Comparison of the nutritional status of VPI with SGA between the EUGR and the non-EUGR groups in the hospital

Variable	Non-EUGR *n* = 84	EUGR *n* = 49	*t/Z/χ*^*2*^	*P*
Start time of enteral feeding h [M (Q1, Q3)]	21.75(3,38)	36(16,90)	2.403	0.016
The amount of milk added with HMF ml [M (Q1, Q3)]	88(60.50,91.50)	100(78,109.60)	2.348	0.019
Time needed to reach the full amount of fortification d [M (Q1, Q3)]	3(3,4.5)	9(3,10)	3.927	< 0.001
Fasting days during hospitalization d [M (Q1, Q3)]	2(0.95,6)	5.9(2,8.10)	3.882	< 0.001
Age of reaching total enteral nutrition d [M (Q1, Q3)]	27(21,35.50)	33(28,50)	3.542	< 0.001
Parenteral nutrition days d [M (Q1, Q3)]	25(16.50,31)	32(23,47)	3.739	< 0.001
Accumulation of amino acids in the first week (g/kg) [M (Q1, Q3)]	17.4(15.20,19.45)	17(14.10,19.60)	0.795	0.426
Accumulation of fat emulsion in the first week g/kg [*x* ± *s*]	13.62 ± 3.94	12.91 ± 5.17	0.827	0.413
Accumulated calories in the first week kcal/kg [*x* ± *s*]	494.78 ± 105.62	461.65 ± 113.63	1.696	0.092
Time for the total calorie to reach 110 kcal/(kg d) d [M (Q1, Q3)]	9.5(7,14)	14(10,22)	3.255	0.001
Time for oral calorie to reach 110 kcal/(kg.) d [M (Q1, Q3)]	27(18.50,33.50)	32(26,45)	3.416	0.001
Maximum physiological weight loss % [M (Q1, Q3)]	5(0.40,7.80)	6(2,8.70)	1.191	0.234
The date of recovery of birth weight d [M (Q1, Q3)]	7(3,9.5)	9(7,12)	2.904	0.004
GV g/kg·d [*x* ± *s*]	18.97 ± 4.77	14.58 ± 2.26	7.16	< 0.001

*EUGR *Is extrauterine growth retardation, *SGA* Is smaller than gestational age, *VPI* Is very premature. G*V* Is growth velocity

#### In-hospital complications of VPI with SGA

Following the criterion of ΔZ of weight at discharge < –1.28, the incidences of hsPDA, NEC stage 2, LOS, and FI in the EUGR group were significantly higher than that in the non-EUGR group (*P* < 0.05). The incidences of complications such as NRDS, EOS, BPD, NEC stage 3, PVL, ROP, PNAC, and MBDP were not significantly different between the groups (*P* > 0.05); see Table 4**.**

**Table 4:** Comparison of the complications related to the hospitalization of VPI with SGA between the EUGR and the non-EUGR groups

Variable	Non-EUGR *n* = 84	EUGR *n* = 49	*χ2*	*P*
NRDS [n (%)]	68(80.95)	37(75.51)	0.551	0.458
hsPDA [n (%)]	37(44.05)	32(65.31)	5.602	0.018
EOS [n (%)]	14(16.67)	6(12.24)	0.474	0.491
FI [n (%)]	35(41.67)	30(61.22)	4.737	0.031
LOS [n (%)]	7(8.33)	11(22.45)	5.269	0.022
NEC ≥stage 2 [n (%)]	4(4.76)	10(20.41)	8.044	0.005
Operation NEC [n (%)]	2(2.38)	2(4.08)	/	0.625
BPD [n (%)]	45(53.57)	33(67.35)	2.422	0.122
NEC ≥ grade 3 [n (%)]	0(0.00)	2(4.08)	/	0.134
PVL [n (%)]	3(3.57)	0(0.00)	/	0.297
ROP requiring intervention [n (%)]	32(38.10)	17(34.69)	0.154	0.695
MBDP [n (%)]	4(4.76)	4(8.16)	/	0.466
PNAC [n (%)]	13(15.48)	10(20.41)	0.526	0.468

Remarks:/:Fisher’s accurate test, no such value

*SGA* is small for gestational age, *VPI* Is extremely premature, *EUGR* Is extrauterine growth retardation, *NRDS* Is neonatal respiratory distress syndrome, *HsPDA* Is patent ductus arteriosus with hemodynamic changes, *EOS* is early-onset sepsis, *FI* Feeding intolerance, *LOS* Is late-onset sepsis, *NEC* Is necrotizing enterocolitis, *BPD* Is bronchopulmonary dysplasia, *IVH* Is intraventricular hemorrhage, *ROP* Is retinopathy of prematurity, *PVL* Is leukomalacia of ventricles, *MBDP* Is a metabolic bone disease of prematurity, *PNAC* Is parenteral nutrition-related cholestasis

### Multivariate logistic regression analysis of EUGR in VPI with SGA

Table 5 presents the results of the multivariate logistic regression analysis, revealing that birth weight, high GV, and male sex were identified as protective factors against EUGR. Conversely, a prolonged duration to achieve complete fortification, slow recovery of birth weight, and NEC stage 2 or higher were identified as independent risk factors for EUGR.

**Table 5:**
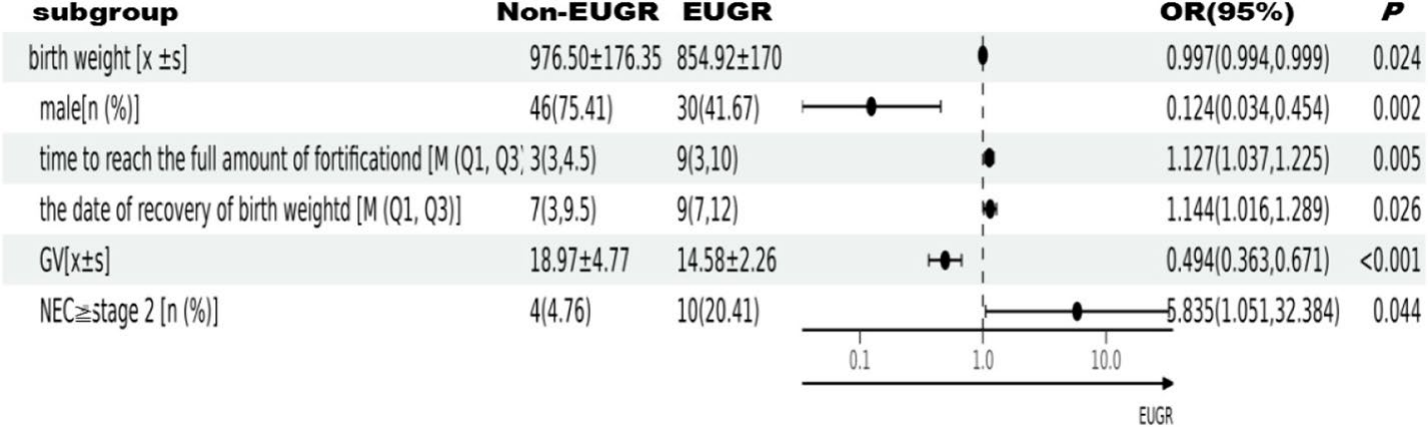
Multivariate logistic regression analysis of EUGR in VPI with SGA

*SGA* Is small for gestational age, *VPI* Is very premature, *EUGR* Is extrauterine growth retardation, *GV* Is growth velocity, *NEC* Is necrotizing enterocolitis

## Discussion

Clark [24] first proposed the concept of EUGR in 2003. He plotted a growth curve to evaluate the incidence of EUGR. However, there are still many controversies about the timing and standard of EUGR evaluation, leading to differences in clinical recommendations and practice [25]. The Fenton curve, which is the revised growth curve for different sexes published in 2013, was established using data from four million premature infants. This comprehensive dataset included information from developed countries such as Germany, Italy, the United States, Australia, Canada, and Scotland, spanning the years 1991 to 2007. The Fenton curve serves as a valuable tool for monitoring and assessing the growth and development of premature infants. According to the data on the gestational age, weight, height, and head circumference of newborns, the accurate *p*-value and the standardized Z value [2] associated with the growth curve of the current growth of newborns can be calculated. This is the most commonly used method to evaluate the intrauterine and extrauterine growth of premature infants. Birth weight serves as a widely adopted indicator for the clinical assessment of newborn growth and nutritional status due to its simplicity, accurate measurement, and reliable repeatability. In clinical practice, the presence of EUGR is typically evaluated based on the weight of premature infants at 36 weeks of corrected gestational age or at hospital discharge. For the same study population, a big difference in the evaluation was found depending on whether the *p*-value or the △Z value on the curve was considered as the criterion. Griffin et al. [26] used two methods to evaluate the incidence of EUGR in 25,899 VPI with a birth weight of 500 ~ 1500 g and gestational age of 22 ~ 32 weeks in California, USA. The incidence of EUGR was 53.3% with the *p*-value of weight at discharge < 10% and 41.4% with △Z value < –1. Premature infants with gestational age ≤ 32 weeks at Mount Sinai Medical Center in the United States were evaluated by Lin et al. [16]. The incidence of EUGR at discharge was found to be 35.3% when using the diagnosis criterion of a discharge weight Z score < –1.28 (equivalent to a *p*-value < 10^th^ percentile). For a △Z (change in Z score) of less than –1.28, the EUGR incidence was 25.5%, and for a △Z of less than –2, the EUGR incidence was 4.5%. There were considerable differences among the three evaluation methods. The incidence of SGA in this cohort was 5.30%, which was slightly lower than the national average [1] and slightly higher than that reported in an American study (4.12%) [27]. In our evaluation of 133 VPI with SGA cases, the incidence of EUGR was 98.50% following the *p*-value criterion and 36.84% following the criterion of △Z < –1.28; there was a discrepancy of 61.66% in this study due to the difference between the evaluated population and the △Z value. The incidence of EUGR differed considerably with different evaluation methods. The *p*-value evaluation method was based on the horizontal evaluation of group data, while the △Z value was based on the vertical evaluation and objective analysis of individual data. Longitudinal evaluation offers a more accurate depiction of the actual growth pattern of neonates [28, 29]. Fenton et al. [30] highlighted shortcomings in the cross-sectional definition itself, emphasizing its limited ability to accurately predict adverse outcomes. The utilization of the 10th percentile as a subjective threshold may result in an overdiagnosis of EUGR, potentially causing parental distress and increasing the risks of overfeeding and obesity. In contrast, the longitudinal definition considers crucial factors such as birth weight and gestational age. It not only helps mitigate the issue of overdiagnosis of EUGR to some extent but also provides a more precise prognosis for preterm infants. Furthermore, in comparison to the cross-sectional definition, the dynamic delta value-based definition demonstrates superior effectiveness in predicting adverse neurodevelopmental outcomes over a 2-year follow-up period [14, 31]. Hence, the delta value-based definition proves to be superior in predicting the long-term outcomes of preterm infants. In our study, we employed the ΔZ value to assess the true incidence of EUGR in VPI with SGA, with the aim of establishing scientific standards for optimizing nutritional strategies for this specific population. Table 1 demonstrates the variations in EUGR diagnosis when different definitions are used, and the application of the longitudinal definition partially mitigated the influence of IUGR. Recently, some researchers have proposed using the lowest postnatal weight age as the reference point for calculating ΔZ value changes. This approach not only offers partial prediction of long-term adverse outcomes but also avoids the impact of physiological postnatal weight loss [32]. Building on this concept, Maiocco et al. [15] conducted a study and revealed that a ΔZ value decrease for head circumference exceeding one standard deviation between discharge and recovery of birth weight within 14 to 21 days after birth is a significant risk factor for neurodevelopmental delays. Unfortunately, this aspect was not considered in the design of our study, and precise evaluation data for EUGR within the 14 to 21-day period were not included in the paper. This limitation provides a direction for future research endeavors.

The results of the univariate analysis showed that the non-EUGR group had a higher birth weight (*P* < 0.001) and a larger Z-value of birth weight (*P* = 0.017). The results of the multivariate analysis showed that high birth weight was a protective factor related to the occurrence of EUGR in VPI with SGA (OR = 0.997, 95% CI: 0.994 ~ 0.999, *P* = 0.024). Our results were similar to those of previous studies [33]. The results showed that the birth weight of infants in the EUGR group was lower, the intrauterine growth was more restricted, and the organs and tissues were relatively underdeveloped. EUGR is caused by scarcity of nutrients in the uterus, greater nutritional demand, and higher energy metabolism, which is more likely to lead to nutritional deficiency and premature infant-related complications after birth [34]. The postnatal nutritional status of VPI with SGA is closely associated with the occurrence of EUGR. The findings from the multivariate analysis indicated that a prolonged duration for breast milk fortification and the slow recovery of birth weight were identified as independent risk factors for EUGR in VPI with SGA, while high GV was found to be a protective factor against EUGR. Breast milk is the best source of nutrition for babies, especially premature infants. However, the energy and nutrients in breast milk cannot meet the growth-related needs of premature infants at the early stages after birth, especially of premature SGA infants. Therefore, HMF containing multiple nutrients is commonly added to breast milk [35].

Our results showed that the quantity of HMF added to milk was more in the EUGR group than that in the non-EUGR group (100 mL vs. 88 mL), and it took longer (9 d vs. 3 d) to reach full fortification in the EUGR group. In China, experts recommend initiating the use of HMF for premature infants when their breastfeeding volume reaches 50–80 mL/(kg·d). It is advised to achieve standard adequate fortification within 3–5 days. A study demonstrated that adding HMF when the breastfeeding volume reaches the recommended threshold was the most effective approach in reducing the incidence of EUGR [36].

In a prospective randomized controlled study conducted by Bozkurt et al. [37], it was observed that achieving full-dose intensive breastfeeding at an earlier stage resulted in higher GV in VPI. This, in turn, contributed to a shorter duration of birth weight recovery. The GV was higher during hospitalization, which was a significant independent protective factor to avoid EUGR and promote the development of the nervous system [38]. Consistent with the findings of this study, Jeffrey et al [39] documented an increase in GV from 11.8 to 12.9 g/kg/day, accompanied by a decrease in the incidence of EUGR in very low birth weight infants (VLBWI) from 64.5% to 50.3%. These results suggested that more attention should be paid to enteral nutrition support for VPI with SGA. By following the recommendations of HMF experts, full breast milk fortification can be achieved at the earliest, the growth rate can be increased, and the recovery time of birth weight can be shortened. These factors play an important role in reducing the incidence of EUGR.

Early postnatal complications directly affect the nutritional supply and extrauterine growth and development of VPI with SGA. The findings from the univariate analysis revealed that the 5-min Apgar score was lower (*P* = 0.012), and the duration of invasive ventilation was longer (*P* = 0.003) in the EUGR group compared to the non-EUGR group. The severity of illness after birth hindered the effective implementation of recommended early enteral nutrition measures, consequently leading to delayed initiation of enteral feeding. The average starting time of enteral feeding of the EUGR group in this study was later than that in the non-EUGR group (36.00 h vs. 21.75 h). A delay in enteral feeding might cause gastrointestinal mucosa atrophy and delayed functional maturity and also increase the incidence of FI (*P* = 0.031) and NEC [40, 41]. The incidence of LOS among infants in the EUGR group was higher than that among infants in the non-EUGR group (*P* = 0.022), which led to longer administration of antibiotics (*P* = 0.011), greater extent of intestinal microecology disorder and a higher incidence of NEC among infants in the EUGR group [42]. The incidence of hsPDA in the EUGR group was higher (*P* = 0.018), the proportion of blood transfusion was higher (*P* = 0.01), and the frequency of blood transfusion was higher (*P* < 0.001) than that in the non-EUGR group. These factors might increase the risk of NEC [43]. In a study, the incidence rate of NEC in premature infants was 2% ~ 5%, among which the incidence rate of very low birth weight infants was 4.5% ~ 8.7% [44]. Our study observed that the incidence of NEC  ≥  stage 2 in the EUGR group was 20.4%. However, no significant difference was found in the occurrence of NEC requiring surgery between the EUGR and non-EUGR groups (*P* = 0.625). The results of the multivariate analysis confirmed that NEC ≥ stage 2 was an independent risk factor for EUGR (OR = 5.835, 95% CI: 1.051–32.384, *P* = 0.044), which showed that the risk of EUGR increased by 5.8 times after NEC occurred in VPI with SGA. These results were similar to those of previous studies [45]. In this study, most infants with NEC ≥ stage 2 were treated conservatively in internal medicine, and clinicians were often very cautious about the fasting time and the indications for re-starting milk, which might lead to a decrease in the nutrient intake [11]. A comprehensive assessment of the risk balance between FI and NEC should be performed to avoid unnecessary fasting and prevent NEC from worsening.

The results of the multivariate analysis also showed that the male sex was a protective factor of EUGR in VPI with SGA. Male infants with premature SGA were reported to have a faster physical catch-up growth in the early postnatal period than female infants [46]. This might be related to the differences in the effects of gender on the physical growth of premature SGA, although it needs to be confirmed in future studies.

## Advantages and limitations

This was the first prospective multicenter study in China to analyze the factors related to the growth pattern of VPI with SGA after birth based on the △Z score. Data were collected from 28 tertiary hospitals in seven regions of China, including general hospitals, children’s hospitals, and women’s and children’s hospitals. While this study did not encompass all very preterm infants in China, it included well-represented tertiary hospitals from diverse regions across the country. Hence, this study provides an objective portrayal of the incidence of EUGR in SGA VPI in China. Our study had some limitations. First, as China is a big country and the data were collected from different hospitals in different regions, the nutrition management strategies among hospitals may differ, leading to differences in the results. Second, as the inclusion criteria excluded cases of death, the correlation between EUGR and the risk of death could not be evaluated. Third, data on VPI with SGA follow-up was lacking, and we aim to conduct a follow-up study on this cohort. In our study, we did not gather data on confounding factors related to the occurrence of EUGR in SGA infants. SGA infants comprise those who are naturally small-sized at birth and those diagnosed with intrauterine growth restriction (IUGR) based on prenatal ultrasound examination. Additionally, IUGR infants may exhibit placental insufficiency, which can increase their vulnerability to both NEC and EUGR. Moreover, other factors like maternal smoking during pregnancy and cumulative postnatal steroid use may introduce biases in the results. Our study primarily focused on diagnosing SGA infants without considering the impact of different etiologies on the occurrence of EUGR in this population. Future research should consider a more comprehensive range of confounding factors and etiologies associated with EUGR in SGA infants to minimize result biases.

## Conclusions

To summarize, using the △Z value to evaluate the occurrence of EUGR in VPI with SGA can more accurately reflect the growth pattern of this special group of infants after birth. The incidence of EUGR following the criterion of △Z value of weight < –1.28 was 36.8%. Regarding VPI with SGA, more attention should be paid to enteral nutrition support. Enhancing enteral nutrition support, attaining complete fortification of breast milk as early as possible, promoting higher GV, reducing the time required for birth weight recovery, and preventing NEC are effective strategies for reducing the incidence of EUGR.

## Data Availability

All data included in this study are available from the correspondence of Xin-Zhu Lin and can be provided upon request as needed.
